# The Diagnostic Yield of Dental Radiography and Cone-Beam Computed Tomography for the Identification of Dentoalveolar Lesions in Cats

**DOI:** 10.3389/fvets.2019.00042

**Published:** 2019-02-21

**Authors:** Colleen M. Heney, Boaz Arzi, Philip H. Kass, David C. Hatcher, Frank J. M. Verstraete

**Affiliations:** ^1^Dentistry and Oral Surgery Service, School of Veterinary Medicine, William Pritchard Veterinary Medical Teaching Hospital, University of California, Davis, Davis, CA, United States; ^2^Department of Surgical and Radiological Sciences, School of Veterinary Medicine, University of California, Davis, Davis, CA, United States; ^3^Department of Population Health and Reproduction, School of Veterinary Medicine, University of California, Davis, Davis, CA, United States; ^4^Diagnostic Dental Imaging Center, Sacramento, CA, United States

**Keywords:** cats, CBCT, cone-beam computed tomography, dental radiography, dentoalveolar lesions, oral diagnostic imaging

## Abstract

The objective of this study was to evaluate the diagnostic yield of dental radiography (DR) and 3 cone-beam computed tomography (CBCT) software modules for the identification of 32 pre-defined dentoalveolar lesions in cats. For 5 feline cadaver heads and 22 client-owned cats admitted for evaluation and treatment of dental disease, 32 predefined dentoalveolar lesions were evaluated separately and scored by use of dental radiography and 3 CBCT software modules [multiplanar reconstructions (MPR), tridimensional (3-D) rendering, and reconstructed panoramic views]. A qualitative scoring system was used. Dentoalveolar lesions were grouped into 14 categories for statistical analysis. Point of reference for presence or absence of a dentoalveolar lesion was determined as the method that could be used to clearly identify the disorder as being present. Accuracy, sensitivity, specificity, and positive and negative predictive values were calculated with the McNemar χ^2^ test of marginal homogeneity of paired data. When all 3 CBCT software modules were used in combination, the diagnostic yield of CBCT was significantly higher than that of dental radiography for 4 of 14 categories (missing teeth, horizontal bone loss, loss of tooth integrity, feline resorptive lesions), and higher, although not significantly so, for 9 categories (supernumerary teeth, supernumerary roots, abnormally shaped roots, vertical bone loss, buccal bone expansion, periapical disease, inflammatory root resorption, and external replacement root resorption). In conclusion, we found that CBCT provided more clinically relevant detailed information as compared to dental radiography. Therefore, CBCT should be considered better suited for use in diagnosing dentoalveolar lesions in cats.

## Introduction

The incorporation of dental radiography (DR) into veterinary dentistry has substantially improved the ability to diagnose dentoalveolar lesions in cats. For 2 decades, full-mouth dental radiography in new feline patients referred for dental treatment has been the diagnostic standard of care given the high diagnostic yield of this imaging modality (1, 2). Cone-beam computed tomography (CBCT) is making inroads into veterinary dental practice, both in terms of adding the third dimension to diagnosis, and in terms of enabling image-guided treatment strategies (3). Market penetration in the human field has been rapid, presumably because CBCT permits a paradigm shift in dental care due to its increased image accuracy, rapid scan time, radiation dose reduction, reduced image artifact, and availability of display modules unique to maxillofacial imaging, compared to conventional CT. Cone-beam computed tomography routinely used in human medicine, has recently become a viable and cost-effective diagnostic alternative that can identify several different types of dentoalveolar lesions in domestic animals (4). Previous studies, as well as part 1 of the present study, have documented its superiority to dental radiography for identifying numerous anatomic structures and dentoalveolar pathologies in domestic species (5–7).

In humans, both CT and CBCT have been shown to be superior in diagnostic accuracy to DR for diagnosing periodontal and endodontic disease (8–11). CBCT has been suggested to be superior in diagnosing periodontitis and endodontic disease in both humans and dogs (8, 12). Experimental studies demonstrating that CBCT imaging is more accurate at diagnosing the severity of periodontitis suggests that DR can both over and under interpret the pathologic features of this disease depending on radiographic technique (4, 8, 11, 13). It is reasonable to presume that CT better delineates alveolar margin height than DR as well. When it comes to endodontic disease, although there is no accepted “gold-standard” for imaging of dentoalveolar lesions in veterinary medicine, the preponderance of human and veterinary literature demonstrating that CT and CBCT imaging modalities are superior to DR for detecting endodontic disease suggests that DR is under-representing the presence of endodontic lesions (12, 14).

Currently in human dentistry, dental radiography is the main method used for the identification of gross lesions and bone loss in the interproximal space (15), evaluation of the periodontal ligament space, lamina dura, and periapical region; and identification of the loss of tooth integrity. In veterinary dentistry, dental radiography currently represents the criterion-referenced standard imaging modality for the evaluation of periodontal and dental health (1, 2, 11). Whenever conventional radiography cannot supply satisfactory diagnostic information for human dentistry, especially for complex cases (i.e., evaluation and treatment of cleft palate, unerupted teeth, or orthognathic surgery), CBCT is regarded as the method of choice (16–18). The use of specialized imaging software designed for processing CBCT images can provide precise sagittal, dorsal, and transverse slices as well as serial transplanar reformation (cross-sections) of each individual tooth, curved planar reformation (simulated distortion-free panoramic images), and indirect volume rendering in tooth and bone modes (19, 20).

The objective of the study reported here was to determine the diagnostic yield of DR as compared to 3 CBCT specialized software modules in the identification of dentoalveolar lesions in cats. We hypothesized that CBCT, specifically serial CBCT slices and multiplanar reconstructions (MPR), would have significantly higher diagnostic yield than dental radiography in the identification of predefined dentoalveolar lesions in cats.

## Materials and Methods

### Animals

Five cadaver heads that were obtained from cats that were euthanized for reasons unrelated to this study were included by design in order to study the software and calibrate the investigators prior to the enrollment of clinical patients. Cats admitted to the Dentistry and Oral Surgery Service at the University of California-Davis for evaluation and treatment of oral disorders between August 2014 and February 2017 for which full-mouth dental radiographs and CBCT scans of the skull were obtained were included in the study. Informed consent was obtained from each client and the study was conducted with approval of the University of California-Davis Institutional Animal Care and Use Committee and the Clinical Trials Review Board.

### Image Acquisition

**Figure 1:**
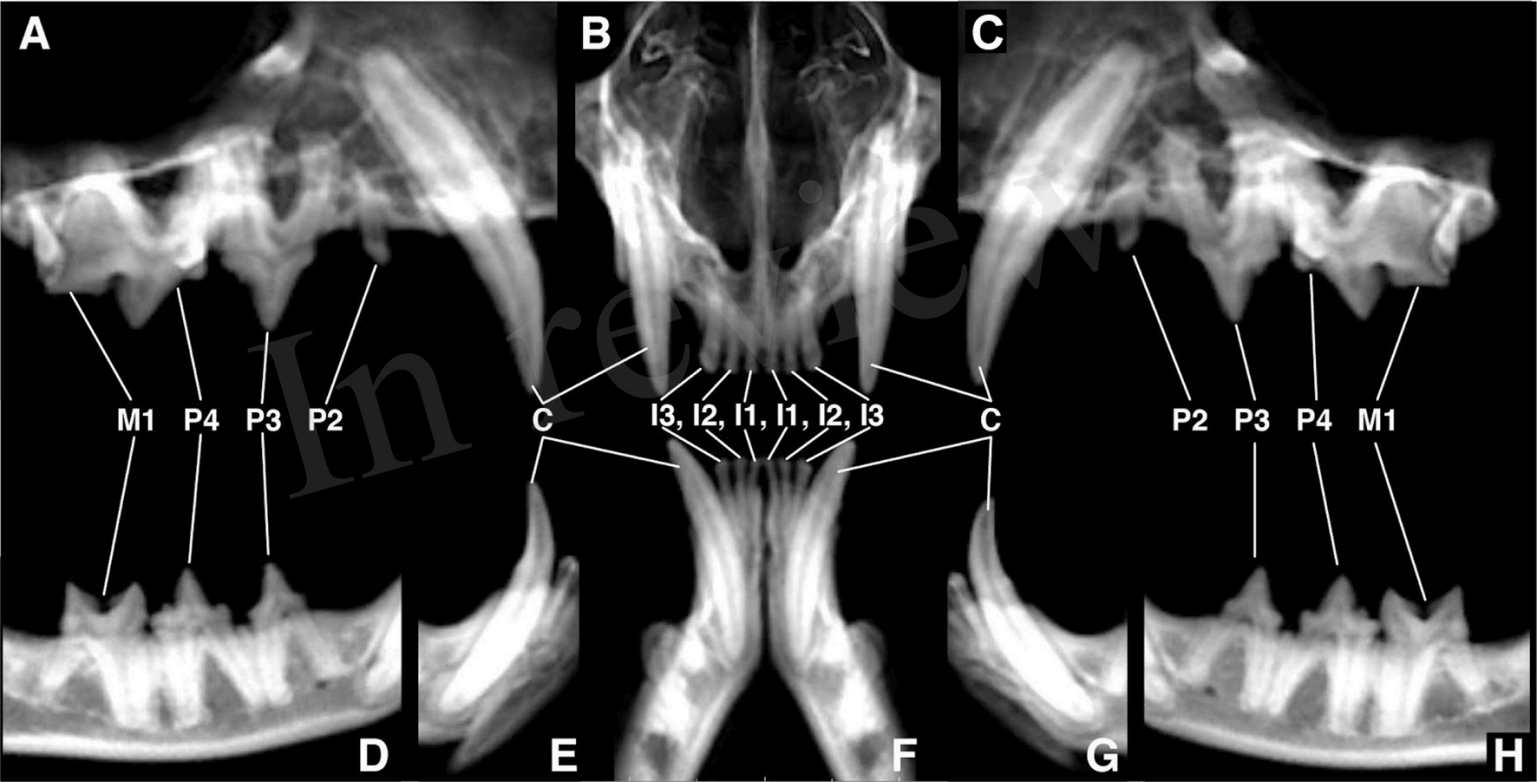
Standardized reconstructed panoramic views for optimal evaluation of dentoalveolar lesions of cats. **(A)** axis of the skull adjusted for orientation of the right maxillary canine tooth through first molar tooth, **(B)** axis of the skull adjusted for orientation of the left and right maxillary first through third incisor teeth, **(C)** axis of the skull adjusted for orientation of the left maxillary canine tooth through first molar tooth, **(D)** axis of the skull adjusted for orientation of the right mandibular third premolar tooth through first molar tooth, **(E)** axis of the skull adjusted for orientation of the right mandibular canine tooth, **(F)** axis of the skull adjusted for orientation of the left and right mandibular first through third incisor teeth, **(G)** axis of the skull adjusted for orientation of the left mandibular canine tooth, **(H)** axis of the skull adjusted for orientation of the left mandibular third premolar tooth through first molar tooth.

Dental radiography and CBCT were performed on the 5 cadaver heads. Client-owned cats were anesthetized, and DR and CBCT were performed. Full-mouth dental radiographs were obtained by use of a digital intraoral imaging system (Heliodent MD, Siemens Sirona; ScanX, Air Techniques) at 60 kVp, 7 mA, and exposure time of 0.12 to 0.20 s (depending on location of the evaluated teeth). This system yielded a resolution of up to 18 line pairs/mm, which equated to a pixel size of 55.5 μm. Radiographic images included the standard series of views in accordance with American Veterinary Dental College guidelines (21). A CBCT unit (NewTom 5G CBCT scanner, NewTom) was used to obtain images. Field of view was 15 x 12 cm, and serial slices of the skull were obtained with a scan time of 18 s, which resulted in a voxel size (slice thickness) of 150 μm. The axis of the skull was modified accordingly to create standardized images for optimal evaluation of the dentition of cats by use of the CBCT reconstructed panoramic views (Figure 1).

### Image Evaluation and Scoring

Dental radiography (DR method) and 3 CBCT specialized software modules [reconstructed panoramic views (Pano method), tridimensional rendering (3-D method), and serial CBCT slices and multiplanar reconstructions (MPR method)] were evaluated separately for their usefulness in identification of 32 predefined dentoalveolar lesions (Table 1). Software manipulation for evaluation of the Pano, 3-D, and MPR methods was performed as described previously (5). Images were examined on medical grade flat-screen monitors (ASUS PB278Q 27-inch, ASUSTeK Computer Inc.) by use of commercially available specialized software (Metron-Dental 7.40.34.0, Epona Tech LCC; Anatomage *Invivo5* dental application; Anatomage Inc.) Each method was scored separately for each dentoalveolar lesion by 1 observer (CH) who was trained and calibrated in image acquisition and interpretation by 2 board-certified veterinary dentists (BA and FJMV) and a board-certified human oral radiologist (DCH).

**Table 1:** Predefined dentoalveolar lesions evaluated in cats by use of DR and CBCT images for each of 3 software modules.

**ANATOMIC AND DEVELOPMENTAL DISORDERS**
Missing tooth
Supernumerary tooth
Supernumerary root
Abnormal eruption: impacted, embedded, rotated
Abnormally shaped root: divergent, convergent, concrescent, dilacerated
PERIODONTITIS*
Vertical bone loss (VBL): mild < 25%, moderate 25% to 50%, severe > 50%
Horizontal bone loss (HBL): mild < 25%, moderate 25% to 50%, severe > 50%
Furcation: involvement, exposure
Buccal bone expansion: mild, moderate, and severe
**TOOTH RESORPTION**
External inflammatory resorption
External root replacement resorption
Feline resorptive lesion
**ENDODONTIC DISEASE**
Loss of tooth integrity: attrition or abrasion, uncomplicated crown fracture (including enamel fracture), complicated crown fracture, uncomplicated crown-root fracture, complicated crown-root fracture, root fracture (including retained tooth roots with a missing coronal segment)
Failure of the pulp cavity to narrow
Periapical lesion

* *VBL < 25% and HBL < 25% = mild periodontitis, which correlates with stage 2 periodontal disease (21); VBL 25% to 50% and HBL 25% to 50% = moderate periodontitis, which correlates with stage 3 periodontal disease (21); and VBL > 50% and HBL > 50% = severe periodontitis, which correlates with stage 4 periodontal disease (21)*.

Whenever possible, dentoalveolar lesions were defined in accordance with nomenclature of the American Veterinary Dental College (22). Qualitative scoring was used for each dental disorder, each imaging modality, and each tooth (0 if the disorder was absent or 1 if the disorder was present). Findings for all 4 methods were recorded separately and without reference to each patient's medical record to limit biased interpretation. After image evaluation was concluded, findings for the DR and CBCT methods were compared to determine a point of reference. The point of reference for presence or absence of a dentoalveolar lesion was determined after completion of image evaluation as the method that could be used to clearly identify the lesions as being present.

The 32 dental lesions were grouped into 14 categories for statistical analysis. Those categories included missing teeth, supernumerary teeth, supernumerary roots, abnormally shaped roots, horizontal bone loss, vertical bone loss, buccal bone expansion, loss of tooth integrity, periapical lesions, inflammatory tooth resorption, external replacement tooth resorption, and feline resorptive lesions. Anatomical and developmental findings were calculated based on a full set of feline dentition (810 teeth total), all other lesions were calculated based on the number of present teeth (789 teeth total).

### Statistical Analysis

Descriptive statistics and mean scores were reported as mean ± *SD*. Discordance of values for the DR and 3 CBCT methods were compared to the point of reference and assessed by use of the McNemar χ^2^ test of marginal homogeneity for paired data. Overall accuracy, sensitivity, specificity, positive predictive value (PPV), and negative predictive value (NPV) were calculated and reported with 95% confidence intervals (CIs). Non-overlapping confidence intervals reflected significant differences between these proportions at the 5% level of significance (*P* < 0.05).

## Results

### Overall Assessment

In addition to the 5 cadaver heads initially evaluated, 22 client-owned cats [17 males (16 castrated and 1 sexually intact) and 5 females (5 spayed and 0 sexually intact)] were included. Breeds included domestic shorthair (*n* = 9), domestic medium hair (2), domestic longhair (3), Burmese (3), Bengal (1), British shorthair (2), Scottish fold (1), and Siamese mix (1). Mean ±*SD* age of the cats was 5.9 ± 3.7 years (range, 5 months to 12 years), and mean body weight was 5.0 ± 1.1 kg (range, 3.3 to 7.2 kg).

The accuracy and PPV of the MPR method was significantly higher compared to the other 3 imaging methods for 4 dentoalveolar lesions (missing teeth, horizontal bone loss, loss of tooth integrity, and feline resorptive lesions). For 4 dentoalveolar lesions (horizontal bone loss, loss of tooth integrity, periapical disease, and feline resorptive lesions), sensitivity of the MPR method was significantly higher than that of the other 3 imaging methods. The accuracy and sensitivity of the DR method was significantly lower than any of the CBCT methods for 1 dentoalveolar lesion (horizontal bone loss). The sensitivity of the MPR method was significantly higher than that of the Pano and 3-D methods, for 1 dentoalveolar lesion (inflammatory root resorption), and was higher, but not significantly so, than that of the DR method. Specificity was significantly higher for the MPR method for 2 dentoalveolar lesions (missing teeth, and horizontal bone loss). The PPV of the Pano method was significantly lower than any of the other 3 imaging methods for 1 dentoalveolar lesion (periapical disease). The NPV was significantly higher for the MPR method for all but 1 dentoalveolar lesion evaluated (missing teeth). For 1 dentoalveolar lesion (horizontal bone loss) the NPV was significantly lower for the DR method compared to any of the other 3 imaging methods.

### Anatomic and Developmental Disorders

Results for the DR and 3 CBCT methods were compared.

#### Missing Teeth

Of the 810 teeth that should have been present according to the dental formula for cats (I 3/3, C 1/1, P 3/2, M 1/1), 24 (2.96%) teeth were missing. Although all the teeth that were truly missing as determined by the point of reference were correctly identified by use of both the DR and MPR methods, 15 additional teeth were incorrectly identified as missing by use of the DR method, when truly root remnants were present. For the Pano method, 18 teeth were falsely identified to be missing. For the 3-D method 14 teeth were thought to be missing. Regardless of the imaging modality, sensitivity and NPV was very high for all methods (100%). Accuracy, specificity, and PPV was significantly higher for the MPR method than for any of the 3 other methods (Table 2).

**Table 2:** Ability to identify missing teeth in 27 cats by use of dental radiography (DR method) and 3 CBCT software modules*.

	**DR**		**PANO**		**3D**		**MPR**	
**Variable**	**Estimated value**	**95% CI**	**Estimated value**	**95% CI**	**Estimated value**	**95% CI**	**Estimated value**	**95% CI**
Accuracy	98.15	96.96–98.96	97.78	96.51–98.68	98.30	97.17–99.07	100	99.55–100
Sensitivity	100	85.75–100	100	85.75–100	100	85.75–100	100	85.75–100
Specificity	98.09	96.87–98.93	97.71	96.40–98.64	98.22	97.03–99.02	100	99.53–100
PPV	61.54	49.22–72.54	57.14	45.79–67.79	63.16	50.49–74.24	100	85.75–100
NPV	100	99.52–100	100	99.52–100	100	99.52–100	100	99.53–100

*Values reported are percentages*.

* *The 3 modules were as follows: reconstructed panoramic views (PANO method), tridimensional rendering (3-D method), and multiplanar reconstructions (MPR method). Differences in variables are significant (P > 0.05) for a method if the CI does not overlap with that of other methods*.

#### Supernumerary Teeth

**Figure 2:**
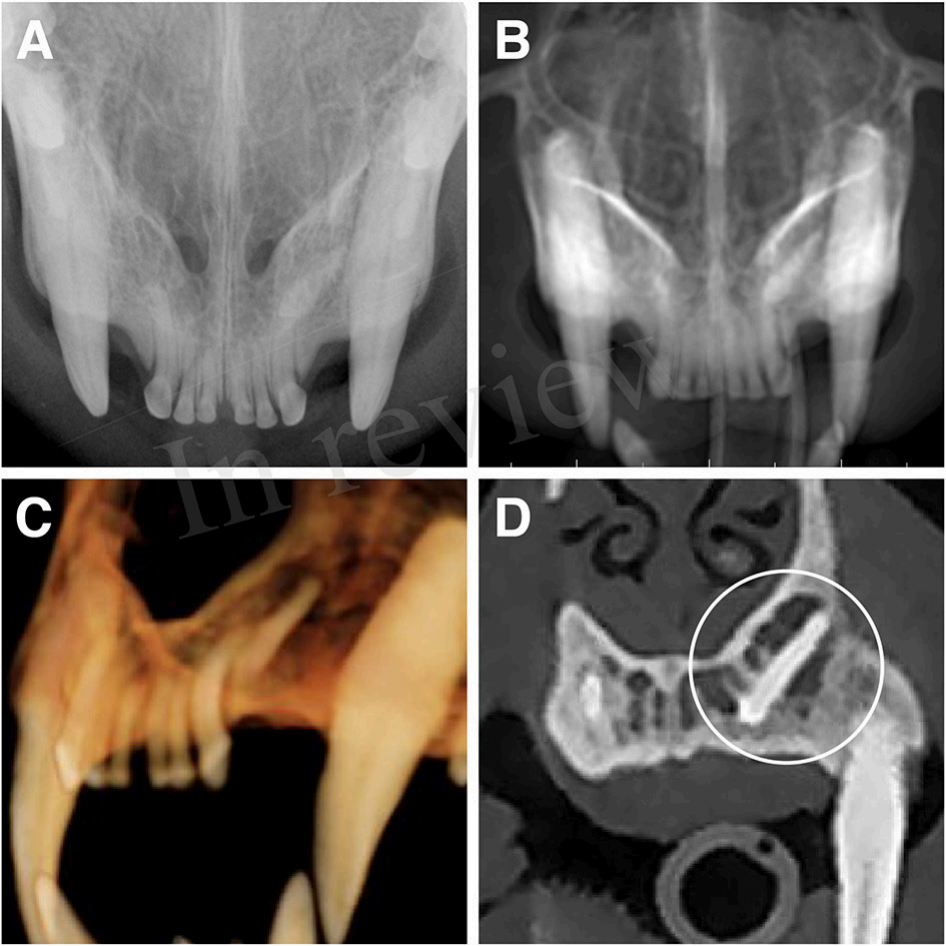
Supernumerary left maxillary third incisor tooth with abnormal eruption. A supernumerary left maxillary incisor tooth was identified as impacted within the left incisive bone. This dentoalveolar lesion was only clearly identified via the MPR method. **(A)** maxillary occlusal dental radiographic view, **(B)** reconstructed panoramic view with the axis of the skull adjusted for orientation of the left and right maxillary first through third incisor teeth, **(C)** tridimensional (3-D) rendering in tooth mode of the left incisive region, **(D)** custom cross-section multiplanar reconstruction decisively showing the supernumerary impacted left maxillary third incisor tooth (circle).

Two instances of erupted supernumerary teeth (supernumerary left and right maxillary second premolar teeth in the same cat) were identified by all 4 imaging modalities. Thus, no statistical analysis was performed on this data. A third supernumerary tooth, which was identified as a supernumerary left maxillary third incisor tooth, was identified via the MPR method only. Thus, no statistical analysis was performed on this data. This tooth was determined to be impacted within the incisive bone and was the only example of abnormal eruption identified (Figure 2).

#### Supernumerary Roots

**Figure 3:**
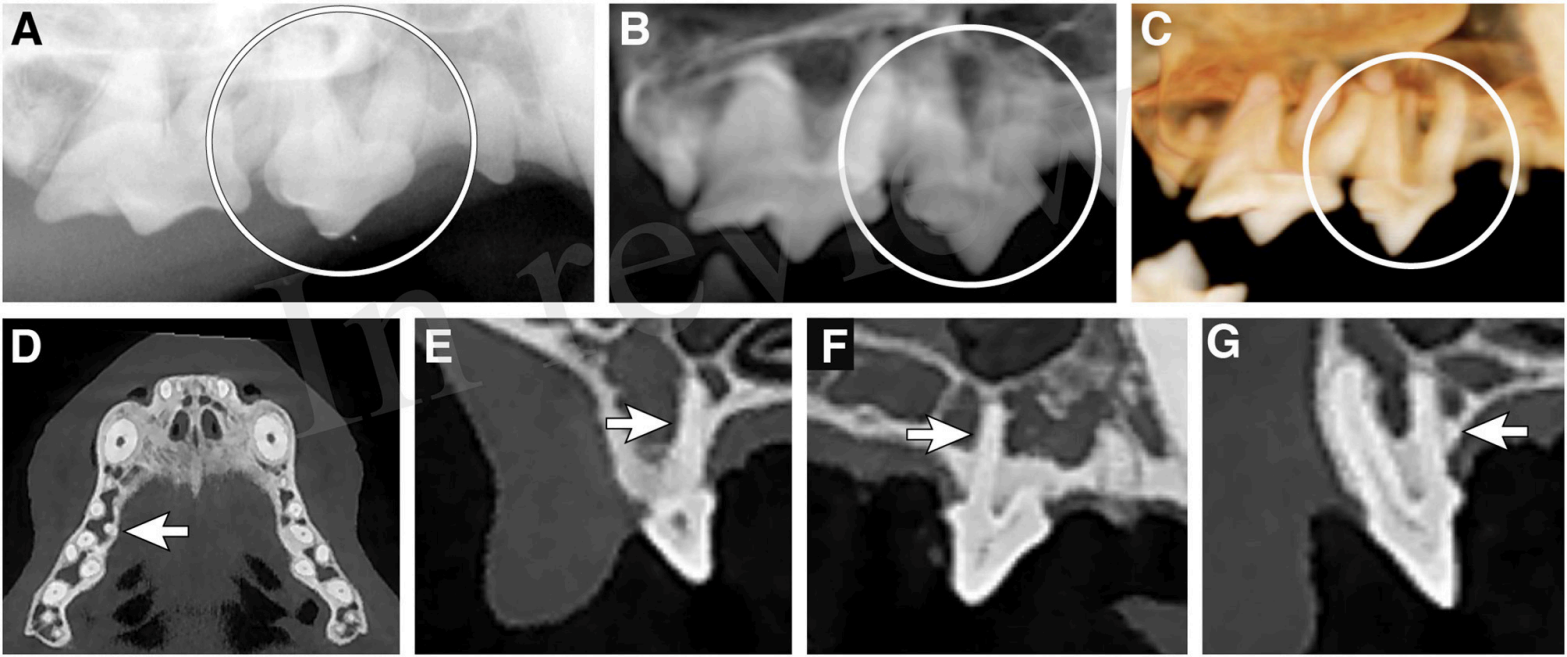
Supernumerary root. Evaluation of a right maxillary fourth premolar tooth supernumerary root. **(A)** Dental radiograph of right maxillary second premolar tooth through first molar tooth obtained with the extraoral, near parallel radiographic technique, **(B)** reconstructed panoramic view with the axis of the skull adjusted for orientation of the right maxillary canine tooth through first molar tooth, **(C)** tridimensional (3-D) rendering in tooth mode of the right maxillary premolar and molar teeth, **(D)** axial section multiplanar reconstruction showing apex of the supernumerary root of the right maxillary third premolar tooth, **(E)** coronal section multiplanar reconstruction showing the supernumerary root of the right maxillary third premolar tooth, **(F)** sagittal section multiplanar reconstruction showing the supernumerary root of the right maxillary third premolar tooth, **(G)** custom cross-section multiplanar reconstruction showing the supernumerary root of the right maxillary third premolar tooth.

4 (0.51%) of 789 possible teeth had a supernumerary root. All four supernumerary roots were associated with the maxillary third premolar teeth (Figure 3). All teeth with an abnormal number of roots were correctly identified only by use of the MPR method. None of the supernumerary roots were identified by any other methods. The difference in accuracy and sensitivity was higher, although not significantly so, for the MPR method over the other 3 methods. The NPV was significantly higher for the MPR method compared to the other 3 methods (Table 3).

**Table 3:** Ability to identify supernumerary roots in cats by use of dental radiography (DR method) and 3 CBCT software modules*.

	**DR**		**PANO**		**3D**		**MPR**	
**Variable**	**Estimated value**	**95% CI**	**Estimated value**	**95% CI**	**Estimated value**	**95% CI**	**Estimated value**	**95% CI**
Accuracy	99.49	98.71–99.86	99.49	98.71–99.86	99.49	98.71–99.86	100	99.53–100
Sensitivity	0.00	0.00–60.24	0.00	0.00–60.24	0.00	0.00–60.24	100	39.76–100
Specificity	100	99.53–100	100	99.53–100	100	99.53–100	100	99.53–100
PPV	NE	NE	NE	NE	NE	NE	100	39.76–100
NPV	99.49	99.49–99.49	99.49	99.49–99.49	99.49	99.49–99.49	100	99.54–100

*Values reported are percentages. Not estimable (NE)*.

* *The 3 modules were as follows: reconstructed panoramic views (PANO method), tridimensional rendering (3-D method), and multiplanar reconstructions (MPR method). Differences in variables are significant (P > 0.05) for a method if the CI does not overlap with that of other methods*.

#### Abnormally Shaped Roots

4 (0.51%) of 789 teeth had abnormally shaped roots (1 was dilacerated, 2 were convergent, and 1 was thickened). All 4 abnormally shaped roots were associated with maxillary third or fourth premolar teeth. The incidences of convergent roots were both distal and mesial buccal roots of maxillary fourth premolar teeth and were identified by all 4 imaging modalities. An additional 2 teeth with abnormally shaped roots (a maxillary fourth premolar tooth with a dilacerated mesial buccal root, and a maxillary third premolar tooth with an abnormally thickened distal root), were identified by the MPR method only. Accuracy and sensitivity for the MPR method was higher, although not significantly so, compared to the other 3 methods. Specificity and PPV was comparable between all 4 methods (100%). NPV was significantly higher for the MPR method (Table 4).

**Table 4:** Ability to identify abnormally shaped roots in cats by use of dental radiography (DR method) and 3 CBCT software modules*.

	**DR**		**PANO**		**3D**		**MPR**	
**Variable**	**Estimated value**	**95% CI**	**Estimated value**	**95% CI**	**Estimated value**	**95% CI**	**Estimated value**	**95% CI**
Accuracy	99.75	99.09–99.97	99.75	99.09–99.97	99.75	99.09–99.97	100	99.53–100
Sensitivity	50	6.76–93.24	50	6.76–93.24	50	6.76–93.24	100	39.76–100
Specificity	100	99.53–100	100	99.53–100	100	99.53–100	100	99.53–100
PPV	100	15.81–100	100	15.81–100	100	15.81–100	100	51.01–100
NPV	99.75	99.33–99.90	99.75	99.33–99.90	99.75	99.33–99.90	100	99.54–100

*Values reported are percentages*.

* *The 3 modules were as follows: reconstructed panoramic views (PANO method), tridimensional rendering (3-D method), and multiplanar reconstructions (MPR method). Differences in variables are significant (P > 0.05) for a method if the CI does not overlap with that of other methods*.

### Periodontal Findings

**Figure 4:**
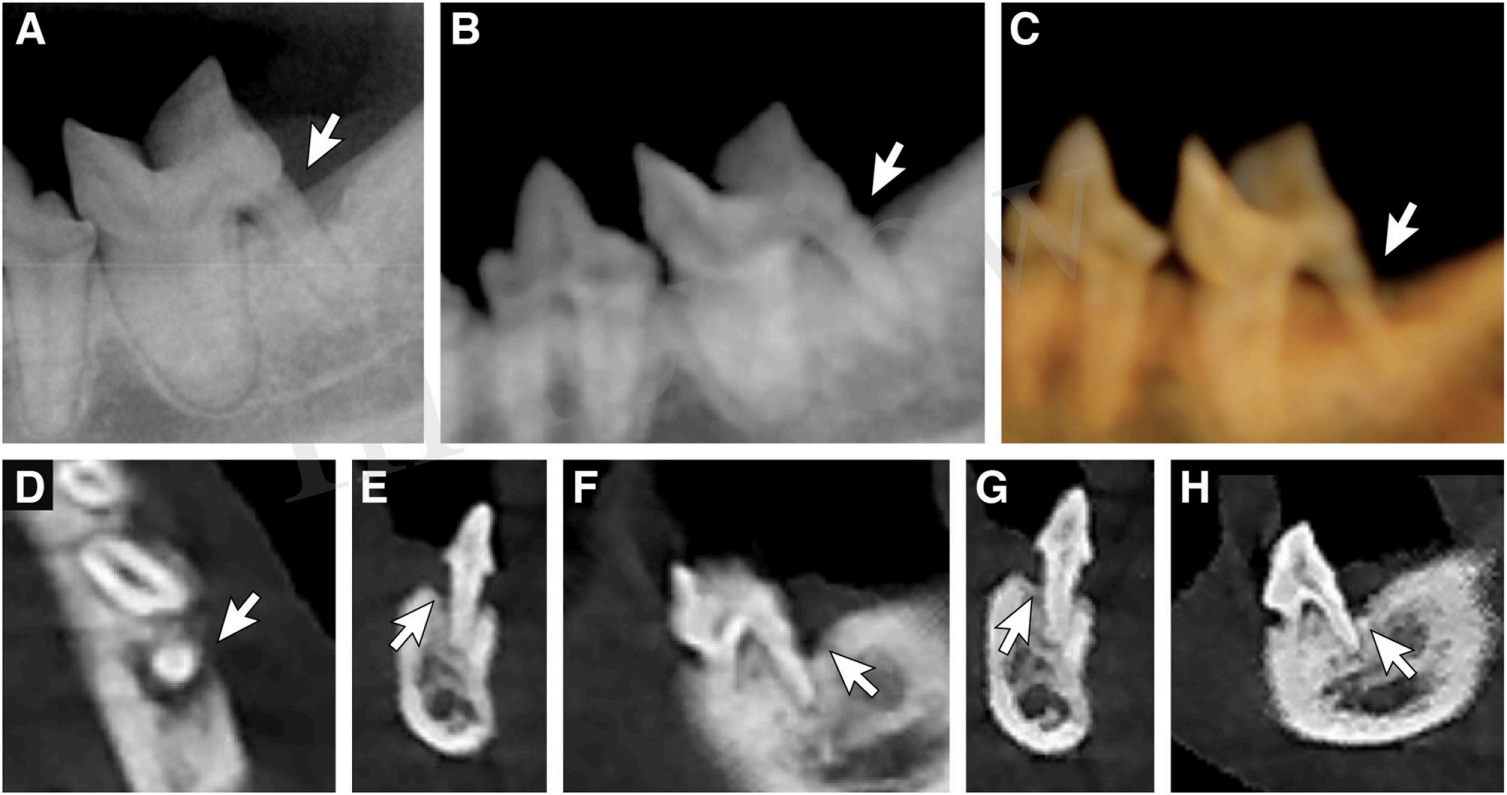
Periodontitis. Evaluation of horizontal and vertical bone loss affecting the distal aspect of the left mandibular first molar tooth by way of **(A)** a dental radiograph obtained with the intraoral, parallel radiographic technique, **(B)** the reconstructed panoramic CBCT viewing method, **(C)** tridimensional rendering CBCT viewing method in tooth mode, **(D)** axial section multiplanar reconstruction showing mid-body of the distal root, **(E)** coronal section multiplanar reconstruction of the distal root, **(F)** sagittal section multiplanar reconstruction of the distal root, **(G,H)** custom cross-section multiplanar reconstructions of the distal root.

Overall, periodontitis was the most prevalent finding (Figure 4). Of the 789 teeth that were present, 359 (45.50%) were affected by alveolar bone loss attributable to periodontal disease [19 (2.40%) teeth with mild horizontal or vertical bone loss; 51 (6.46%) teeth with moderate horizontal or vertical bone loss, furcation involvement, or both; and 289 (36.63%) teeth with severe horizontal or vertical bone loss, furcation exposure, or both). Compared with the point of reference, the extent of combined horizontal and vertical bone loss was under-interpreted for 194 teeth with the DR method, 111 teeth with the Pano method, and 111 teeth with the 3-D method, and it was overinterpreted for 17 teeth with the DR method, 19 teeth with the Pano method, and 19 teeth with the 3-D method.

The teeth most commonly affected by severe periodontitis were the mandibular incisor teeth (*n* = 98) and the maxillary canine teeth (*n* = 37). These teeth coincided with the areas of maximum crowding and represented the sites of the biggest disagreement among diagnoses between the DR and MPR methods.

#### Horizontal Bone Loss

Of the 789 teeth that were present, 357 (45.24%) were affected by horizontal bone loss attributable to periodontal disease [19 (2.40%) teeth with mild horizontal bone loss; 50 (6.34%) teeth with moderate horizontal bone loss; and 288 (36.50%) teeth with severe horizontal bone loss]. Compared with the point of reference, the extent of horizontal bone loss was under interpreted for 194 teeth with the DR method, 111 teeth with the Pano method, and 111 teeth with the 3-D method, and it was over interpreted for 17 teeth with the DR method, 19 teeth with the Pano method, and 19 teeth with the 3-D method. The MPR method had significantly higher accuracy (99.99%), sensitivity (97.76%), specificity (100%), PPV (100%), and NPV (98.81%) compared to any of the other 3 imaging methods. The MPR method under-interpreted the degree of horizontal bone loss in only 8 of 357 instances (Table 5). The DR method had the significantly lowest accuracy (73.26%), sensitivity (73.95%), and NPV (69.14%) compared to all other imaging methods.

**Table 5:** Ability to identify horizontal bone loss in cats by use of dental radiography (DR method) and 3 CBCT software modules*.

	**DR**		**PANO**		**3D**		**MPR**	
**Variable**	**Estimated value**	**95% CI**	**Estimated value**	**95% CI**	**Estimated value**	**95% CI**	**Estimated value**	**95% CI**
Accuracy	73.26	70.02–76.32	83.52	80.75–86.05	83.52	80.75–86.05	99.99	98.01–99.56
Sensitivity	45.66	40.41–50.98	68.91	63.82–73.67	68.91	63.82–73.67	97.76	95.63–99.03
Specificity	96.06	93.77–97.69	95.60	93.22–97.33	95.60	93.22–97.33	100	99.15–100
PPV	90.56	85.58–93.93	92.83	89.24–95.28	92.83	89.24–95.28	100	98.95–100
NPV	69.14	66.00–70.21	78.82	76.10–81.30	78.82	76.10–81.30	98.18	96.46–99.08

*Values reported are percentages*.

* *The 3 modules were as follows: reconstructed panoramic views (PANO method), tridimensional rendering (3-D method), and multiplanar reconstructions (MPR method). Differences in variables are significant (P > 0.05) for a method if the CI does not overlap with that of other methods*.

#### Vertical Bone Loss

Of the 789 teeth that were present, 24 (3.04%) were affected by vertical bone loss attributable to periodontal disease [none with mild vertical bone loss; 2 (0.25%) teeth with moderate vertical bone loss; and 22 (2.79%) teeth with severe vertical bone loss]. Compared with the point of reference, the extent of vertical bone loss was under-interpreted for 9 teeth with the DR method, 13 teeth with the Pano method, and 15 teeth with the 3-D method, and it was never over interpreted by any method. The MPR method had significantly higher accuracy (99.87%) compared to all the other 3 imaging methods and under-interpreted the degree of vertical bone loss in only one instance (Table 6). The MPR method had higher, although not significantly, sensitivity (95.83%) and NPV (99.87%) compared to the DR method. All 4 imaging methods had a comparable specificity and PPV.

**Table 6:** Ability to identify vertical bone loss in cats by use of dental radiography (DR method) and 3 CBCT software modules*.

	**DR**		**PANO**		**3D**		**MPR**	
**Variable**	**Estimated value**	**95% CI**	**Estimated value**	**95% CI**	**Estimated value**	**95% CI**	**Estimated value**	**95% CI**
Accuracy	98.86	97.85–99.48	98.35	97.20–99.12	98.10	96.88–98.93	99.87	99.30–100
Sensitivity	62.50	40.59–81.20	45.83	25.55–67.18	37.50	18.80–59.41	95.83	78.88–99.89
Specificity	100	99.52–100	100	99.52–100	100	99.52–100	100	99.52–100
PPV	100	78.20–100	100	71.51–100	100	66.37–100	100	85.18–100
NPV	98.84	98.07–99.30	98.35	97.20–99.12	98.08	97.40–98.58	99.87	99.12–99.98

*Values reported are percentages*.

* *The 3 modules were as follows: reconstructed panoramic views (PANO method), tridimensional rendering (3-D method), and multiplanar reconstructions (MPR method). Differences in variables are significant (P > 0.05) for a method if the CI does not overlap with that of other methods*.

#### Buccal Bone Expansion

Of the 107 canine teeth that were present in the study, 19 had buccal bone expansion [5 (4.67%) teeth with mild buccal bone expansion; 8 (7.48%) teeth with moderate mild buccal bone expansion; and 6 (5.61%) teeth with severe mild buccal bone expansion]. Compared with the point of reference, the extent of buccal bone expansion was under-interpreted for 4 teeth with the DR method, 3 teeth with the Pano method, and 10 teeth with the 3-D method, and it was never overinterpreted (Table 7).

**Table 7:** Ability to identify buccal bone expansion in cats by use of dental radiography (DR method) and 3 CBCT software modules*.

	**DR**		**PANO**		**3D**		**MPR**	
**Variable**	**Estimated value**	**95% CI**	**Estimated value**	**95% CI**	**Estimated value**	**95% CI**	**Estimated value**	**95% CI**
Accuracy	96.26	90.70–98.97	97.20	92.02–99.42	90.65	83.48–95.43	100	96.61–100
Sensitivity	78.95	54.43–93.95	84.21	60.42–96.62	47.37	24.45–71.14	100	82.35–100
Specificity	100	95.89–100	100	95.89–100	100	95.89–100	100	95.89–100
PPV	100	78.20–100	100	79.41–100	100	66.37–100	100	82.35–100
NPV	95.65	90.21–98.13	96.70	91.22–98.81	89.90	85.17–93.09	100	95.94–100

*Values reported are percentages*.

* *The 3 modules were as follows: reconstructed panoramic views (PANO method), tridimensional rendering (3-D method), and multiplanar reconstructions (MPR method). Differences in variables are significant (P > 0.05) for a method if the CI does not overlap with that of other methods*.

### Endodontic Disease

Of the 789 teeth that were present, 51 (6.46%) had signs of endodontic disease. Endodontic disease was characterized by loss of tooth integrity (*n* = 45), periapical lesion (*n* = 6), or a combination of the two (*n* = 1; a root fracture of the left mandibular third incisor tooth with periapical lesion).

#### Loss of Tooth Integrity

**Figure 5:**
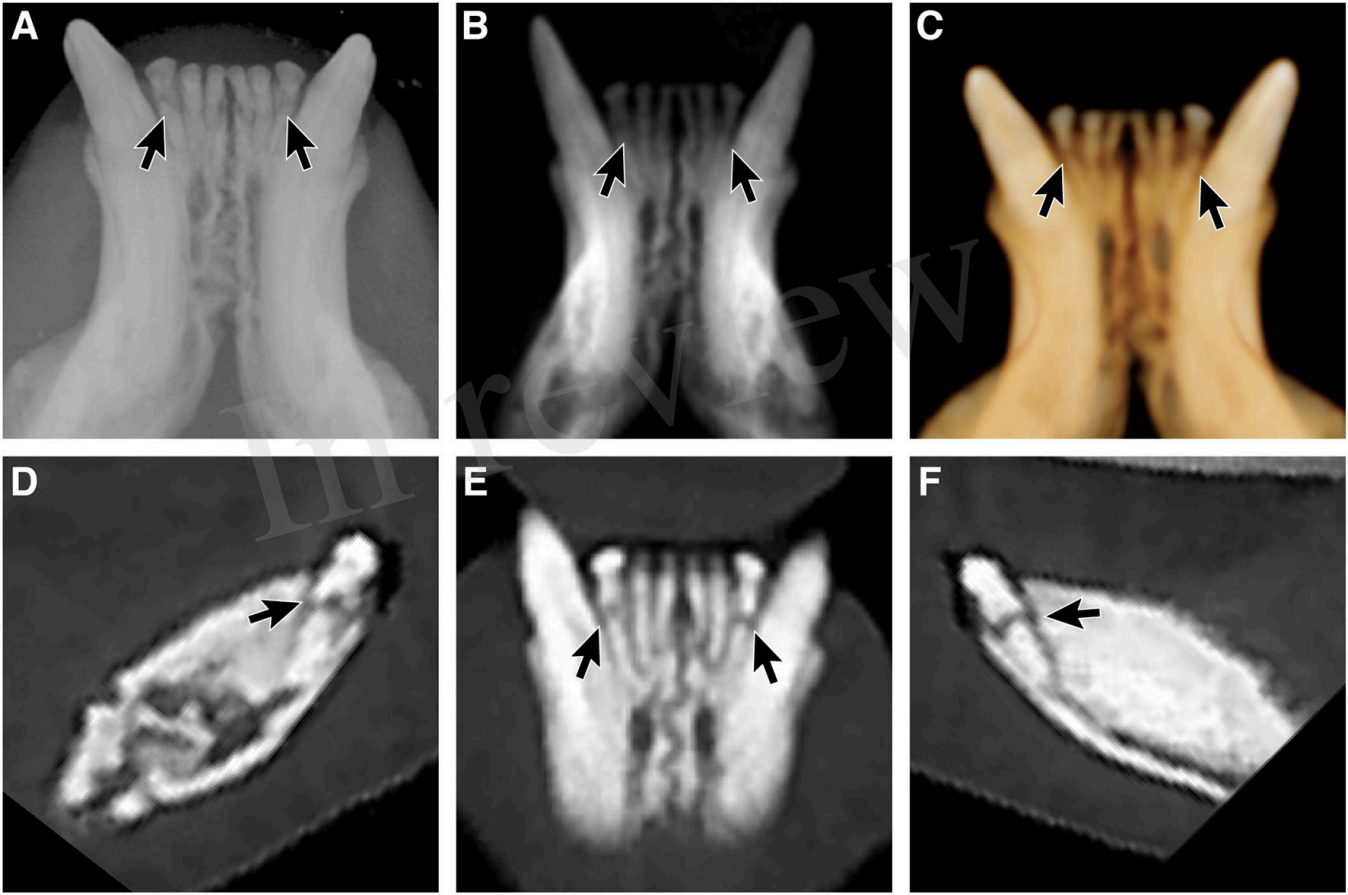
Root fractures. Evaluation of the integrity of the left and right mandibular third incisor teeth demonstrating root fractures by way of **(A)** a mandibular occlusal radiographic view, **(B)** reconstructed panoramic CBCT viewing method with the axis of the skull adjusted for orientation of the left and right mandibular first through third incisor teeth, **(C)** tridimensional (3-D) rendering CBCT viewing method in tooth mode, **(D)** custom cross-section multiplanar reconstruction of the right mandibular third incisor tooth**, (E)** custom cross-section multiplanar reconstruction displaying the root fractures of both the left and right mandibular third incisor teeth, **(F)** custom cross-section multiplanar reconstruction of the left mandibular third incisor tooth.

Of the 789 teeth that were present, 45 (5.70%) had loss of integrity of the crown or root. Of these, 2 were determined radiographically to be uncomplicated crown fractures, whereas 43 teeth were determined to have root fractures (Figure 5). The MPR method was the only method able to detect all of these findings with a significantly higher accuracy, sensitivity, PPV, and NPV (Table 8). The DR method missed 21 (46.67%) teeth with root fractures, and falsely identified 1 uncomplicated crown fracture and 2 additional root fractures. The Pano method missed 23 (51.11%) teeth with root fractures, and falsely identified 1 additional root fracture. The 3-D method missed 20 (44.44%) with loss of tooth integrity (2 uncomplicated crown fractures and 18 root fractures), and falsely identified 2 additional root fractures. The Pano method had the lowest sensitivity, although not significantly so, for detecting loss of tooth integrity (48.89%).

**Table 8:** Ability to identify loss of tooth integrity in cats by use of dental radiography (DR method) and 3 CBCT software modules*.

	**DR**		**PANO**		**3D**		**MPR**	
**Variable**	**Estimated value**	**95% CI**	**Estimated value**	**95% CI**	**Estimated value**	**95% CI**	**Estimated value**	**95% CI**
Accuracy	96.96	95.51–98.04	96.96	95.51–98.04	97.21	95.81–98.24	100	99.55–100
Sensitivity	53.33	37.87–68.34	48.89	33.70–64.23	55.56	40.00–70.36	100	92.13–100
Specificity	99.60	98.83–99.92	99.87	99.25–100	99.73	99.03–99.97	100	99.52–100
PPV	88.89	71.45–96.24	95.65	75.21–99.38	92.59	75.35–98.08	100	92.13–100
NPV	97.24	96.27–97.97	97.00	95.51–98.04	97.38	96.40–98.09	100	99.52–100

*Values reported are percentages*.

* *The 3 modules were as follows: reconstructed panoramic views (PANO method), tridimensional rendering (3-D method), and multiplanar reconstructions (MPR method). Differences in variables are significant (P > 0.05) for a method if the CI does not overlap with that of other methods*.

#### Failure of the Pulp Cavity to Narrow

No incidences of failure of the pulp cavity to narrow were identified by comparison with the contralateral tooth in the same position of the dental quadrant when present for any tooth by any of the 4 imaging methods.

#### Periapical Lesion

**Figure 6:**
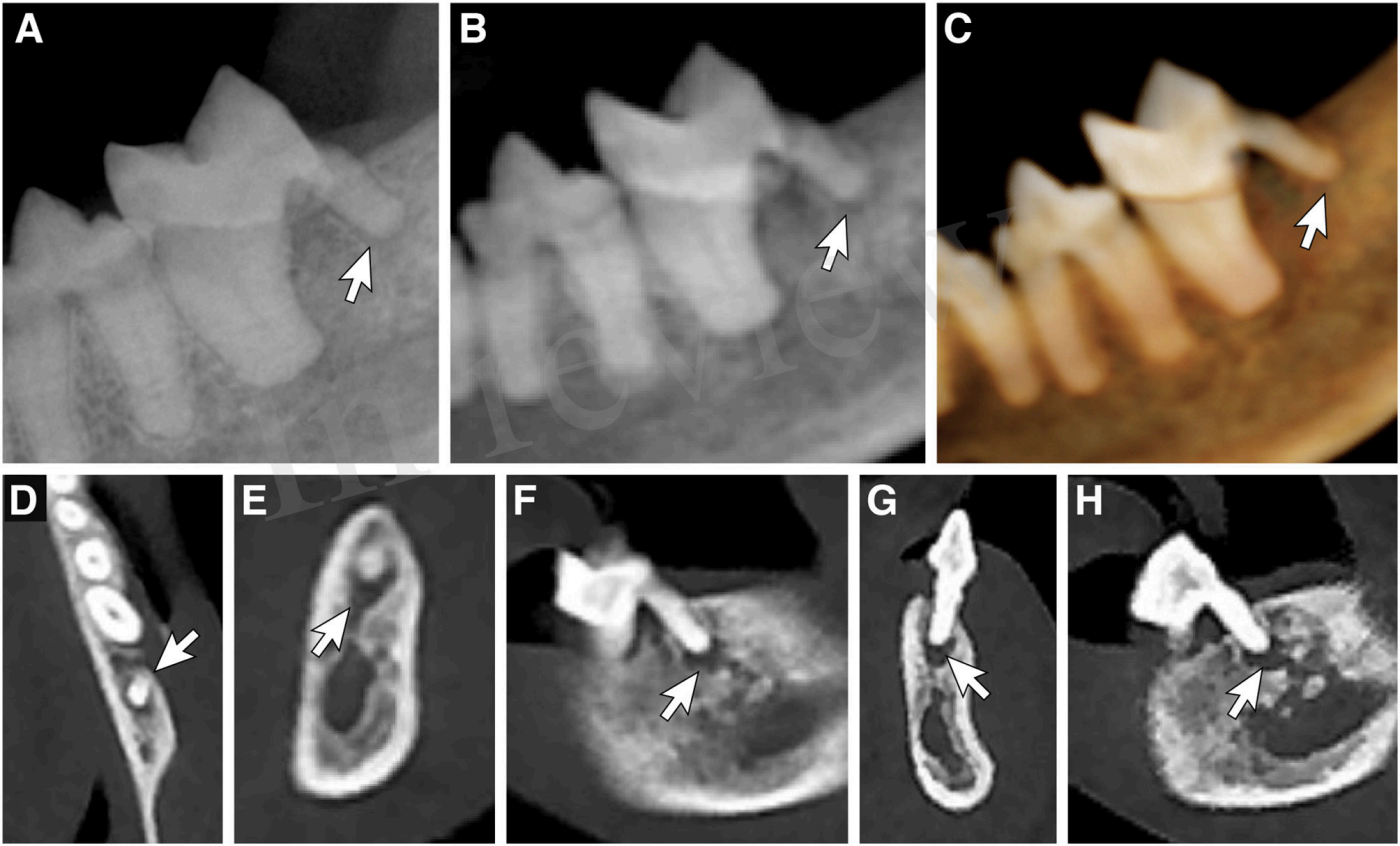
Periapical lesion. Evaluation of the presence of a periapical lesion associated with the distal root of the left mandibular first molar tooth by way of **(A)** a dental radiograph obtained with the intraoral, parallel radiographic technique, **(B)** reconstructed panoramic CBCT viewing method with the axis of the skull adjusted for orientation of the left mandibular third premolar tooth through first molar tooth, **(C)** tridimensional (3-D) rendering CBCT viewing method in tooth mode, **(D)** axial section multiplanar reconstruction showing apex of the distal root, **(E)** coronal section multiplanar reconstruction of the distal root, **(F)** sagittal section multiplanar reconstruction of the distal root, **(G,H)** custom cross-section multiplanar reconstructions of the distal root.

Periapical disease was associated with 7 of 789 (0.89%) teeth (a total of 4 maxillary fourth premolar teeth, 2 mandibular first molar teeth, and a single first incisor tooth were affected) (Figure 6). All lesions could be identified only by use of the MPR method. Six (85.71%) teeth were missed by all the other 3 imaging methods. The Pano method falsely identified 1 periapical lesion (associated with a mandibular first molar tooth). The sensitivity and NPV of the MPR method was significantly higher than the other 3 methods (Table 9). The MPR method had a higher, although not significantly so, accuracy. The Pano method had a PPV that was significantly lower than any of the other 3 imaging methods. The DR, Pano, and 3-D methods all had comparably low sensitivity (14.29%) for periapical disease. The DR, 3-D, and MPR methods all had comparably high specificity and PPV for periapical disease.

**Table 9:** Ability to identify periapical disease in cats by use of dental radiography (DR method) and 3 CBCT software modules*.

	**DR**		**PANO**		**3D**		**MPR**	
**Variable**	**Estimated value**	**95% CI**	**Estimated value**	**95% CI**	**Estimated value**	**95% CI**	**Estimated value**	**95% CI**
Accuracy	99.24	98.35–99.72	99.11	98.18–99.64	99.24	98.35–99.72	100	99.53–100
Sensitivity	14.29	0.36–57.87	14.29	0.36–57.87	14.29	0.36–57.87	100	59.04–100
Specificity	100	99.53–100	99.87	99.29–100	100	99.53–100	100	99.53–100
PPV	100	2.50–100	50.00	6.48–93.52	100	2.50–100	100	59.04–100
NPV	99.24	98.97–99.44	99.24	98.97–99.44	99.24	98.97–99.44	100	99.54–100

*Values reported are percentages*.

* *The 3 modules were as follows: reconstructed panoramic views (PANO method), tridimensional rendering (3-D method), and multiplanar reconstructions (MPR method). Differences in variables are significant (P > 0.05) for a method if the CI does not overlap with that of other methods*.

### Tooth Resorption

Results for the methods are compared. Tooth resorption was defined in accordance with American Veterinary Dental College Guidelines (23). For lesions that could not be classified by these guidelines, inflammatory root resorption was defined as internal or external root resorption seen associated with periodontitis or endodontic disease, and external root replacement resorption was defined as loss of the periodontal ligament without loss of tooth substance (24, 25).

#### Inflammatory Root Resorption

Of 789 teeth, 20 (2.53%) were affected by inflammatory root resorption. All lesions were detected in 8 of 27 cats. Two cats had 12 teeth affected (6 teeth each). The most frequently affected teeth were the mandibular first through third incisor teeth (9), followed by the mandibular canine teeth (3). Detection of all teeth affected by inflammatory root resorption was only achieved by use of the MPR method. The NPV of the MPR method was significantly higher than that of the other 3 methods (Table 10). The accuracy, sensitivity, specificity, and PPV of the MPR method was higher, but not significantly so, than the DR, Pano, and 3-D methods. Seven teeth with inflammatory root resorption were missed by the DR method, 9 were missed with the Pano method, and 12 were missed with the 3-D method.

**Table 10:** Ability to identify inflammatory root resorption in cats by use of dental radiography (DR method) and 3 CBCT software modules*.

	**DR**		**PANO**		**3D**		**MPR**	
**Variable**	**Estimated value**	**95% CI**	**Estimated value**	**95% CI**	**Estimated value**	**95% CI**	**Estimated value**	**95% CI**
Accuracy	99.11	98.18–99.64	98.86	97.85–99.49	98.52	97.42–99.23	100	99.53–100
Sensitivity	65.00	40.78–84.61	55.00	31.53–76.94	40.00	19.12–63.95	100	83.16–100
Specificity	100	99.52–100	100	99.52–100	100	99.52–100	100	99.52–100
PPV	100	75.29–100	100	71.51–100	100	63.06–100	100	83.16–100
NPV	99.10	98.37–99.50	98.84	98.14–99.28	98.50	97.87–98.95	100	99.53–100

*Values reported are percentages*.

* *The 3 modules were as follows: reconstructed panoramic views (PANO method), tridimensional rendering (3-D method), and multiplanar reconstructions (MPR method). Differences in variables are significant (P > 0.05) for a method if the CI does not overlap with that of other methods*.

#### External Root Replacement Resorption

External root replacement resorption was associated with 2 (0.25%) of 789 teeth (a mandibular second incisor tooth and mandibular third premolar tooth). Neither of these teeth were diagnosed with external root replacement resorption by the DR, Pano, or 3-D methods. The NPV of the MPR method was significantly higher than that of the other 3 methods (Table 11). The accuracy, sensitivity, specificity, and PPV of the MPR method was higher, but not significantly so, than the DR, Pano, and 3-D methods.

**Table 11:** Ability to identify root replacement resorption in cats by use of dental radiography (DR method) and 3 CBCT software modules*.

	**DR**		**PANO**		**3D**		**MPR**	
**Variable**	**Estimated value**	**95% CI**	**Estimated value**	**95% CI**	**Estimated value**	**95% CI**	**Estimated value**	**95% CI**
Accuracy	99.75	99.09–99.97	99.75	99.09–99.97	99.75	99.09–99.97	100	99.53–100
Sensitivity	0.00	0.00–84.19	0.00	0.00–84.19	0.00	0.00–84.19	100	15.81–100
Specificity	100	99.53–100	100	99.53–100	100	99.53–100	100	99.53–100
PPV	NE	NE	NE	NE	NE	NE	100	15.81–100
NPV	99.75	99.75–99.75	99.75	99.75–99.75	99.75	99.75–99.75	100	99.54–100

*Values reported are percentages. Not estimable (NE)*.

* *The 3 modules were as follows: reconstructed panoramic views (PANO method), tridimensional rendering (3-D method), and multiplanar reconstructions (MPR method). Differences in variables are significant (P > 0.05) for a method if the CI does not overlap with that of other methods*.

#### Feline Resorptive Lesions

**Figure 7:**
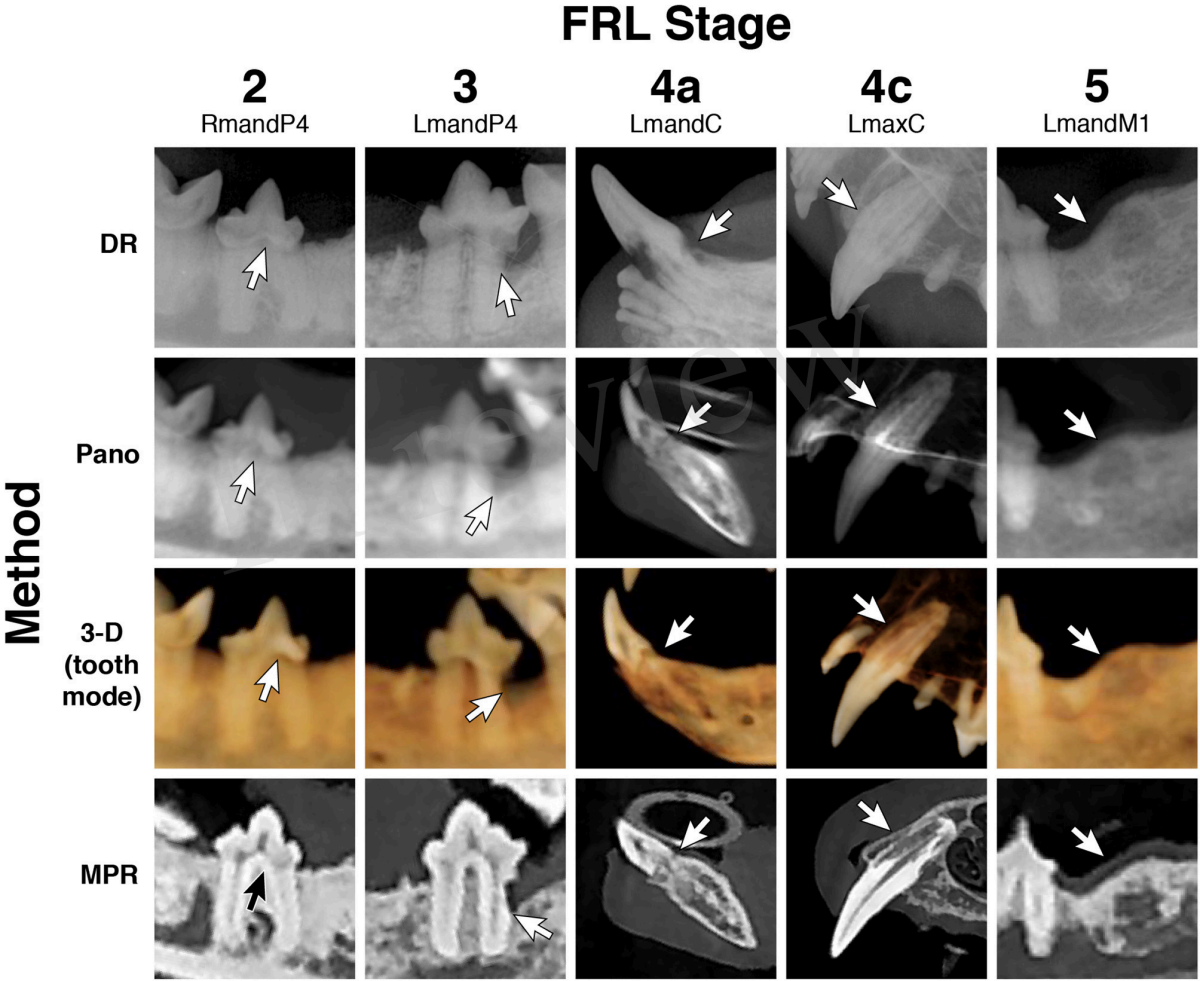
Feline resorptive lesions. Representative images obtained by use of the DR method, Pano method, 3-D method in tooth mode, and custom cross-section MPR method, and used for the evaluation of various radiographic stages of feline resorptive lesions (stages 2, 3, 4a, 4c, and 5). The white arrows indicate the dentoalveolar lesion identified.

Of 789 teeth, 97 (12.30%) were affected by feline resorptive lesions (Figure 7). All lesions were detected in 10 of the 27 cats. Three cats had 62 affected teeth (19, 21, and 22 teeth). The most frequently affected teeth were the mandibular third premolar teeth (15), followed by the mandibular first molar teeth (11), and the maxillary third premolar teeth (10). There were no incidences of any of the mandibular incisor teeth being affected by feline resorptive lesions. Detection of all affected teeth was obtained only by the MPR method. The accuracy, sensitivity, PPV, and NPV of the MPR method were significantly higher than all other methods (Table 12). The specificity was also higher, although not significantly so, for the MPR method over the DR, Pano, and 3-D methods. The sensitivity of the DR, Pano, and 3-D methods were found to be very low (56.70, 59.79, and 57.73 respectively). Forty-two teeth with feline resorptive lesions were missed by the DR method, 39 were missed with the Pano method, and 41 were missed with the 3-D method. Additionally, the DR and 3-D methods both falsely diagnosed feline resorptive lesions in 1 and 2 teeth respectively.

**Table 12:** Ability to identify feline resorptive lesions in cats by use of dental radiography (DR method) and 3 CBCT software modules*.

	**DR**		**PANO**		**3D**		**MPR**	
**Variable**	**Estimated value**	**95% CI**	**Estimated value**	**95% CI**	**Estimated value**	**95% CI**	**Estimated value**	**95% CI**
Accuracy	94.55	92.73–96.03	95.06	93.30–96.46	94.55	92.73–96.03	100	99.53–100
Sensitivity	56.70	46.25–66.73	59.79	49.35–69.63	57.73	47.28–67.70	100	96.27–100
Specificity	99.86	99.20–100	100	99.47–100	99.71	98.96–99.96	100	99.47–100
PPV	98.21	88.50–99.75	100	93.84–100	94.39	93.02–95.50	100	96.27–100
NPV	94.27	92.91–95.38	94.66	93.30–95.77	94.55	93.22–95.63	100	99.48–100

*Values reported are percentages*.

* *The 3 modules were as follows: reconstructed panoramic views (PANO method), tridimensional rendering (3-D method), and multiplanar reconstructions (MPR method). Differences in variables are significant (P > 0.05) for a method if the CI does not overlap with that of other methods*.

## Discussion

To our knowledge, this is the first study to investigate the diagnostic yield of CBCT compared to DR for the evaluation of dentoalveolar lesions in cats. In the present study, we found several important and clinically relevant differences and similarities in diagnostic yield between imaging methods. First, the DR method frequently diagnosed teeth as “missing” when root fracture fragments were present. Second, the MPR method appears to be more sensitive for the detection of periapical lesions in cats. Importantly, dentoalveolar lesions with low frequency of occurrence were identified by the MPR method more reliably than any other imaging method. Additionally, MPR was the only method that identified the presence of supernumerary roots of the maxillary third premolar teeth. Finally, the overall diagnostic yield of CBCT is significantly higher than DR for the identification of feline resorptive lesions.

Dental radiography frequently diagnosed teeth as “missing” when root fracture fragments were present. This agrees with previous reports in human dentistry that documented increased ability for the identification of deciduous roots or unerupted teeth by use of CBCT (14, 26). The higher accuracy for the MPR method in the diagnosis of root fractures in the present study has been supported by findings for several human endodontic studies (27–33). A limitation of the DR method is that the root fracture line can only be delineated if the x-ray beam passes directly through it. Due to this, there is a risk in misdiagnosing the presence of a root fracture with the use of dental radiography because of the possibility of an oblique course of the fracture line in the sagittal plane. The DR, Pano, and 3-D methods had a low PPV for the ability to identify a tooth as truly missing, and this finding supports that the MPR method is the most appropriate method for evaluating apparently missing teeth. This finding is of significant clinical importance, as the presence of unidentified tooth roots can be a cause for oral pain. Additionally, retained tooth roots could potentially lead to failure of clinical improvement or clinical resolution of feline chronic gingivostomatitis following treatment with full mouth extractions (34, 35).

MPR appears to be more sensitive, although not significantly so, for the detection of periapical lesions in cats. Periapical lesions have been shown to occur with very low prevalence in cats, with a previous study diagnosing un-expected periapical lesions in 3.5% of cats by use of dental radiography (1). Due to the low prevalence of this type of lesion in cats, this study failed to determine statistical significance in the difference in diagnostic yield between imaging modalities. Two studies of dogs (36, 37) which compared findings for periapical radiography with CBCT findings and used histopathological evidence as the diagnostic criterion-referenced standard, revealed that use of dental radiography detected fewer periapical lesions than did use of CBCT, and underestimated their size. A complex background pattern, or too much unaffected mass on either side of the root apex may be the reason that teeth with periapical lesions that were undetected by the DR method were found by the MPR method. Additionally, false negative diagnosis made by the DR method may have been the results of lack of a well-defined border of the lesions. This finding is of clinical importance as it shows that the absence of radiolucency with the DR method does not guarantee a healthy periapex and utilizing the DR method for the detection of periapical lesions should be done with care due to the increased possibility of a false-negative diagnosis. Additionally, in clinical cases of oral pain of unknown origin, it should be kept in mind that having no radiographic findings of endodontic disease does not guarantee that endodontic disease is not present, and the use of CBCT for evaluation of periapical health should be considered.

Dentoalveolar lesions with low frequency of occurrence (supernumerary roots, abnormally shaped roots, periapical lesions, inflammatory root resorption, external replacement root resorption) were identified by the CBCT imaging modality, specifically by the MPR software module, more reliably than by the DR imaging modality. The value of CBCT for use in the evaluation of tooth and root canal morphology in humans has recently been reported (38–43). Use of CBCT as a diagnostic method for the evaluation of tooth resorption has been validated in human dentistry (44–48). A diagnostic modality utilized for the evaluation of diseases with low prevalence should have high sensitivity. The DR method was determined to have relatively poor sensitivity for the detection of dentoalveolar lesions with low prevalence in this study. In situations where patients have poorly localized symptoms associated with an untreated or previously treated tooth, and clinical and dental radiographic examination show no evidence of disease, CBCT may reveal the presence of previously undiagnosed pathology. CBCT may also be indicated to help confirm the absence of an odontogenic etiology for pain when managing non-odontogenic causes of pain.

MPR was the only method that definitively identified supernumerary roots of the maxillary third premolar teeth. In a skull study, prevalence of a supernumerary root on the maxillary third premolar was found to be 10.3% (49), whereas in a radiographic study (1) the prevalence of supernumerary roots in total was 1.7%. This corresponds with the occurrence of false negative diagnoses for supernumerary roots of the maxillary third premolar tooth by the DR method seen in this study. For the assessment of teeth with 3 roots, CBCT enabled 3-D reconstruction along with the evaluation of all roots and the tooth itself from all view angles and dimensions. A previous study comparing CBCT with panoramic radiography for reliability in identifying roots of mandibular third molar teeth in humans supports this finding (50). To perform the appropriate radiographic technique to acquire optimal imaging by the DR method of a 3 rooted tooth, not only does the bisecting angle between the tooth and film need to be appropriately identified, but the focus must be turned in a mesial-distal direction to acquire a clear image and avoid superimposition of the supernumerary buccal and palatine roots Custom MPR slice angles can be chosen so that the frontal and sagittal slices, respectively, become parallel with the longitudinal axis of the root and, therefore, the axial slices perpendicular to it. These factors make the superiority of CBCT imaging over conventional dental radiography obvious. This has clinical implications for surgical planning for the extraction of these teeth and supports the necessity for accurate diagnosis of supernumerary roots in feline patients, especially for those that undergo full mouth extractions for treatment of feline chronic gingivostomatitis.

Diagnostic yield of CBCT is significantly higher than DR for the identification of feline resorptive lesions. Lesions on the buccal or lingual aspect of a tooth cannot always be clearly identified by the DR method (51). 3-D reconstruction, tooth visualization from all angles, and minimum image distortion resulted in a more reliable diagnosis of FRLs by the CBCT modality, which is consistent with a previous study (4). Clinical studies in humans have demonstrated that conventional dental radiography grossly underestimates certain resorptive lesions, such as inflammatory root resorption when compared with CBCT (52). Of relevance is the previously reported ability of CBCT to detect simulated resorption cavities with minimal dimensions, which were representative of early lesions. Two human dental studies (45, 53) independently demonstrated that CBCT was significantly better than dental radiography at identifying small, artificial root resorption cavities, with dimensions as little as 0.5 x 0.25 mm and 0.3 and 0.15 mm, respectively. These studies highlight the potential ability of CBCT to detect incipient feline resorptive lesions before it becomes identifiable by the DR method. A future study could look at agreement between tactile examination (dental charting), DR, and CBCT for the diagnosis of FRLs.

Periodontitis is generally considered the most common dental disease of cats (54). The lack of differentiation between the buccal and lingual alveolar margin with the DR method is one important aspect for potentially underestimating the amount of bone loss, especially for infra-alveolar bony pockets (9). In a study in which infra-alveolar bony defects were experimentally produced and measured utilizing dental radiography and high-resolution computed tomography, it was found that there was an average underestimation of 0.6 mm in dental radiographs and an overestimation of 0.2 mm in high resolution conventional computed tomography. This might explain the reason that findings for the MPR method commonly coincided with the point of reference because the bone level was easily detectable around the entire tooth or within the furcation area of teeth with multiple roots. Studies that have been conducted to compare the use of 2-D and 3-D images for identifying artificial bone defects (11, 55) revealed that dental radiography has a sensitivity of 63–67%, whereas CBCT has a sensitivity of 80–100%. The CBCT imaging modality allows for the mesio-distal and bucco-lingual dimension to easily be identified so that all infra-alveolar bony defects can be classified by the number of surrounding walls into one-, two-, and three-walled bony defects (56). This is clinically relevant, as knowledge about the morphology of the bony pocket is an important component for the prognosis and surgical treatment planning of strategic teeth, because a higher number of walls around a periodontal bone defect improve the potential for regeneration of alveolar bone.

The present study revealed that CBCT provided more detailed and more accurate information than dental radiography, thereby making CBCT better suited than dental radiography for the use in diagnosing dentoalveolar disorders in cats. Although the MPR method had perfect scores of 100% for sensitivity, specificity, NPV, and PPV for 9 categories (missing teeth, supernumerary roots, abnormally shaped roots, buccal bone expansion, loss of tooth integrity, periapical disease, inflammatory root resorption, root replacement resorption, and feline resorptive lesions), the 95% CI must be taken into consideration, especially for disorders with a low prevalence, to put these results in context.

Limitations of the study reported here included the lack of histopathological evidence of disease, which is an inherent problem in clinical studies. Additionally, it is possible that certain lesions were missed by both dental radiography and CBCT, thereby falsely elevating or decreasing the reported accuracy of dental radiography, CBCT, or both. Finally, the presence of dentoalveolar lesions identified with CBCT but not with dental radiography (or vice versa) raise the question of validity for either of the diagnostic imaging modalities. The absence of definitive and objective methods (i.e., information in a necropsy report) required standardized viewing methods to be utilized by the primary evaluator. It should be considered that, in a clinical setting, findings on dental radiographs are combined with findings for periodontal probing and dental charting to compensate for some of the shortcomings of dental radiography. However, a comparison of CBCT and dental radiography combined with findings of periodontal probing and dental charting was beyond the scope of the study reported here.

In conclusion, we qualitatively assessed the ability to identify dentoalveolar lesions in cats by use of dental radiography and 3 CBCT software modules. When all 3 software modules were used in combination, the diagnostic yield for CBCT was significantly higher than that for dental radiography in 4 of 14 categories (missing teeth, horizontal bone loss, loss of tooth integrity, feline resorptive lesions), and higher, although not significantly so, in all other dentoalveolar lesion categories. Direct comparison of dental radiography and CBCT revealed that although resolution played an important role, the ability to obtain an unobstructed view of dentoalveolar conditions, as was obtained by use of the MPR method, resulted in a higher diagnostic yield for CBCT, as has been reported in previous study (7). In a clinical setting, the 3-D images and Pano method will help clinicians quickly get an overall impression of dental health and disease. However, the most detailed information can be gained by the MPR method.

## Author Contributions

CH study concept and design; acquisition of data; analysis and interpretation of data; drafting of the manuscript. BA and FV study concept and design; analysis and interpretation of data; critical revision of the manuscript for important intellectual content; administrative, technical, or material support; study supervision; obtaining funding. DH critical revision of the manuscript for important intellectual content. PK statistical analysis.

### Conflict of Interest Statement

The authors declare that the research was conducted in the absence of any commercial or financial relationships that could be construed as a potential conflict of interest.
